# Relationship between CD4 count and quality of life over time among HIV patients in Uganda: a cohort study

**DOI:** 10.1186/s12955-015-0332-3

**Published:** 2015-09-15

**Authors:** Doris Mutabazi Mwesigire, Faith Martin, Janet Seeley, Achilles Katamba

**Affiliations:** Department of Medicine, Makerere College of Health Sciences, PO Box 7072, Kampala, Uganda; Department of Psychology, University of Bath, Bath, United Kingdom; MRC/UVRI Uganda Research Unit on AIDS, Uganda Virus Research Institute, Entebbe, Uganda

## Abstract

**Background:**

Immunological markers (CD4 count) are used in developing countries to decide on initiation of antiretroviral therapy and monitor HIV/AIDS disease progression. HIV is an incurable chronic illness, making quality of life paramount. The direct relationship between quality of life and CD4 count is unclear. The purpose of this study is to determine the relationship between change in CD4 count and quality of life measures in a Ugandan cohort of people living with HIV.

**Methods:**

We prospectively assessed quality of life among 1274 HIV patients attending an HIV clinic within a national referral hospital over a period of 6 months. Quality of life was measured using an objective measure, the Medical Outcomes Study HIV health survey summarized as Physical Health Score and Mental Health Score and a subjective measure, the Global Person Generated Index. Generalized estimating equations were used to analyze the data. The primary predictor variable was change in CD4 count, and the outcome was quality of life scores. We controlled for sociodemographic characteristics, clinical factors and behavioral factors. Twenty in-depth interviews were conducted to assess patient perception of quality of life and factors influencing quality of life.

**Results:**

Of the 1274 patients enrolled 1159 had CD4 count at baseline and six months and 586 (51 %) received antiretroviral therapy. There was no association found between change in CD4 count and quality of life scores at univariate and multivariate analysis among the study participants whether on or not on antiretroviral therapy. Participants perceived quality of life as happiness and well-being, influenced by economic status, psychosocial factors, and health status.

**Conclusions:**

Clinicians and policy makers cannot rely on change in immunological markers to predict quality of life in this era of initiating antiretroviral therapy among relatively healthy patients. In addition to monitoring immunological markers, socioeconomic and psychosocial factors should be underscored in management of HIV patients.

## Background

There is an established relationship between immunological and virological outcomes as important markers of HIV disease progression and treatment failure [1, 2]. CD4 cell count has been reported to have a strong association with progression to AIDS-related illness or death [3]. With the availability of antiretroviral therapy (ART) coupled with early diagnosis, people living with HIV (PLHIV) now live longer. Quality of life (QoL) has become an important outcome variable to be monitored, in addition to other clinical outcomes and biological markers such as CD4 count [4]. With the prolonged survival of HIV patients in resource-limited settings, the focus in HIV care is no longer only on clinical outcomes such as morbidity and mortality but on QoL as well. QoL measurement is now more essential than ever, to optimize patient outcomes [4]. However, it is not clear how QoL and CD4 count are related, with few studies and inconsistent results. Understanding the relationship between immunological response and QoL will improve the effectiveness, receptiveness and accuracy of care and other support services to PLHIV in resource-limited settings.

Some studies have looked at factors associated with QoL, including CD4 count in developed and developing count [5–12]. CD4 count has been associated with QoL in some studies reporting poor QoL with low CD4 count or high QoL with high CD4 count [6, 8, 11–15]. Baseline CD4 count has been found to be a predictor of health-related QoL [14]. Another cross-sectional study reported that improving CD4 count is likely to also improve health-related QoL [15]. More recent studies in Africa have reported a positive association between CD4 count and QoL. A multi-site study in Uganda and Kenya found a positive association between Physical Health Score (PHS) and CD4 count among a mixed population of patients on ART and those not on ART [12] . Related to this, a study in Nigeria found a CD4 count ≥350 cells/μL was associated with better QoL scores in the physical domain [11]. These studies were not designed to systematically evaluate the relationship between measured change in CD4 count and any change in QoL. Most of these studies were cross-sectional and thus could not provide the temporal association between QoL and CD4 count.

The studies that have been carried out to specifically assess the relationship between immunological and virological outcomes and QoL have reported conflicting outcomes. For example, in a randomized double-blind controlled study carried out over a period of 24 months, an association between change in QoL and change in CD4 count was reported, and change in CD4 count was a stronger long-term predictor of QoL compared with change in viral burden [16]. Another study in South Africa assessed the change in CD4 count and viral load among HIV patients not on ART and those that had been on ART for 12 months and found weak correlations between CD4 count and QoL measures [17]. Similar weak correlations and no associations were reported by Venter and colleagues in a cross–sectional study [18]. Bucciardi and colleagues [19] showed no correlation between CD4 count and health-related QoL among ART-experienced patients; this was also reported by Magafu and colleagues among adult Tanzanian patients [20], as well as Arpinelli and colleagues among Italian patients [21]. In India, a mixed picture was reported; some QoL subscales were associated with CD4 count but the majority of subscales were not; associations were only reported in the subgroup with low CD4 count [22]. A positive association between CD4 and emotional well-being was reported in a cohort of men living with HIV at baseline and at 12 months; however, there was no such association with any other QoL subscales at baseline and at 12 months [23]. Interestingly, Call and colleagues [24] found an association with CD4 count and PHS as well as another five out of the eight SF-36 health survey subscale scores. Patients with higher CD4 counts had higher QoL scores; however, there was no association between CD4 count and MHS in this group.

Very few studies in resource-limited settings have assessed immunological markers (CD4 count) and QoL over time, yet immunological markers and QoL change over time [6]. Additionally, the few studies that have assessed the association between QoL and CD4 count worldwide have reported inconsistent results. The aim of this study was to determine the association between change in QoL and change in immunological status (CD4 count) among PLHIV attending an urban clinic in Uganda. While patients are on ART, the CD4 count is expected to increase and hence provide better immunity and, most likely, better QoL. When a person with HIV is not on ART, the CD4 count is expected to decrease with time, and the person would start on ART when eligible. The assumption is that QoL drops with decreased CD4 count. Our study will test this assumption and contribute to the knowledge gap in developing countries, where CD4 count is the immunological marker most used for determining ART initiation and HIV/AIDS disease monitoring. The findings from in-depth interviews will be used to explain the findings from quantitative analysis.

## Methods

### Study participants

A cohort of people living with HIV was drawn from a population of HIV patients attending the HIV clinic at the Mulago National Referral Hospital. The inclusion criteria for the study were adult patients ages ≥18 years; none were on ART at baseline. One group was ready to initiate therapy and started ART on the day of the baseline interview (*n* = 640), and the other group was not eligible for ART (CD4 >350 cells/μL) and received basic care and no ART (*n* = 634). Patients were excluded if they did not consent or were acutely ill and required medical or surgical treatment or admission to the hospital.

### Sample size

After a literature search, we could find no studies comparing change in CD4 count and change in QoL among patients on ART and non-ART patients. We used a longitudinal study in which all patients were on ART and calculated the sample size for the ART group in our study.

Mean PHS at baseline was 49.57 (SD 8.14) and the mean change in PHS per week was 0.09 (SD 0.10). The change in 24 weeks was postulated at 2.16 and hence the mean PHS estimated to be 51.73 [16].

We used PASS 13 software (NCSS, LLC, Kaysville, UT, USA) for sample size calculations. A sample size of 107 achieves 80 % power to detect a difference of −2.2 between the null hypothesis of mean 49.5 and the alternative mean of 51.7. Estimated sample size for one-sample comparison of mean to hypothesized value: a sample size of 150 is required to detect a change in PHS of 2.16 assuming alpha =0.05, power =90 %.

### Study design

**Fig. 1:**
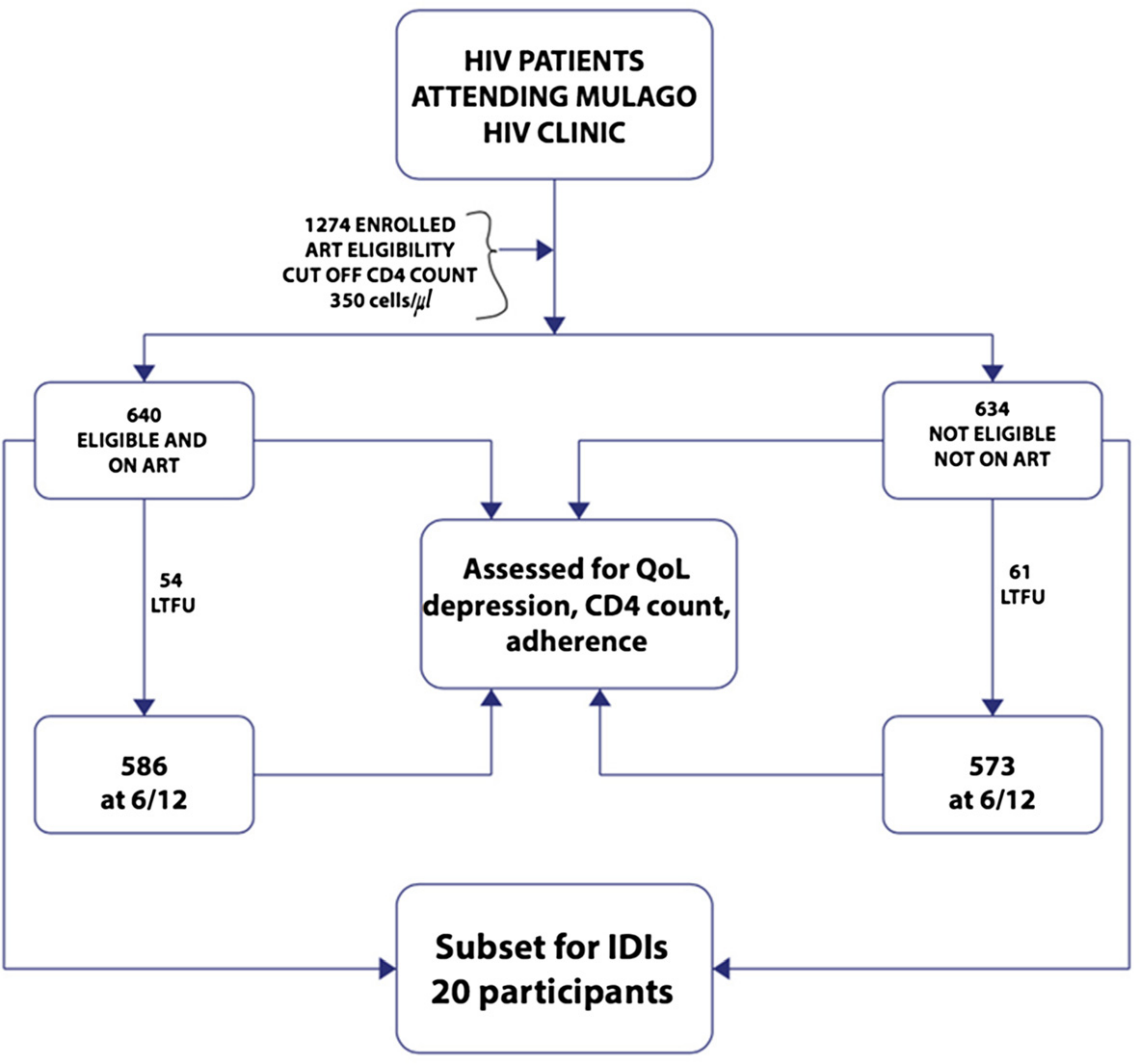
Schematic patient flow chart showing the recruitment and follow up of 1274 study participants at the Mulago HIV clinic in Uganda between April 2012 and December 2013

This was a prospective cohort study. Participants were enrolled consecutively as they registered per clinic day. Enrollment began in April 2012 and was completed in December 2013. World Health Organization (WHO) guidelines were used to initiate ART in patients with a CD4 count less than 350, or co-infected with TB regardless of CD4 count, or co-infected with hepatitis B regardless of CD4 count [25]. We approached 1274 PLHIV and all consented to participate in the study. Of these, 640 initiated ART on the day of the baseline interview. Additionally, 20 in-depth interviews were conducted to understand what this study population perceived as QoL and what influenced their QoL. The 20 interviewees were a purposely selected subset of the total 1274 enrolled in the study; they were chosen to represent differences in age and gender among participants. In addition, 10 of these 20 participants received ART, and 10 were not eligible for ART and received only basic care (no ART). This qualitative study has been described elsewhere [26]. The figure below illustrates the patient recruitment procedure (Fig. 1).

### Data collection

At enrollment, we collected data using a questionnaire and structured interview on sociodemographic characteristics such as age, gender, marital status, socioeconomic status (employment status, income per month and level of education), behavioral characteristics (alcohol consumption (CAGE) and smoking), clinical characteristics (WHO disease stage, antiretroviral therapy (on ART or not), and depression (see below for measure details)), and history of opportunistic infection. Blood was drawn at the baseline visit for CD4 counts. If a CD4 blood test had been done within 14 days before the enrollment day, it was not repeated and the CD4 count was retrieved from the clinic records. At 6 months, blood was drawn for another CD4 count and the results given to the patient on their next clinic visit by their primary care provider. Interviews were conducted in person by the first author and two supervised research assistants at baseline, 3 months and 6 months. To maintain data quality, the first author reviewed a sample of the data collection forms to ensure correctness. Double data entry in Epidata v3.1 was done by the two assistants and the first author checked and compared these data with the original scripts if there were discrepancies. At the end of the study, we reviewed the records of patients lost to follow-up to determine if there were any differences in the reasons for loss between the two groups (ART and non-ART).

### Measures

We used two measures to assess QoL so as to capture the subjective and objective aspects of QoL. The objective measure used was the Medical Outcomes Study HIV (MOS-HIV) health survey and the subjective measure was the Global Person Generated Index (GPGI). The objective measure mainly captures levels of functioning and symptoms and gives a clinician an indication of how the patient is progressing. However, this measure ignores individual patient perception and what may be important to them regarding their QoL. The subjective measure allows the individual to express what their QoL is like [27], thereby providing an understanding of how the individual perceives their QoL.

The MOS-HIV tool is preferred because it is an HIV disease-specific measure and among the most widely used patient-reported outcome measures [28, 29]. It has been validated in Uganda [7, 30, 31] and found to be useful in capturing QoL data in this setting. The validated Luganda version of the MOS-HIV was used in this study. The MOS-HIV has also been proved to be able to differentiate between patients on ART and those that are not on ART, and between symptomatic and asymptomatic HIV patients [32]. It has 10 subscales: physical function, role function, social function, cognitive function, mental health, energy and fatigue, health distress, health perceptions, pain and overall QoL [28, 29]. The subscales are summarized into two summary scores, PHS and MHS, using a recommended mean of 50 (SD 10) [33].

The GPGI score is a subjective measure that has been validated in other developing countries [34]. The GPGI measures global QoL and allows the individual to define and rate their QoL. The individual will therefore define “what” QoL is and “how” QoL is for them [27]. The GPGI is capable of capturing “response shift” [35]. The individual states five areas of their life that are the most important to them and these are scored on a scale of 0–6, where zero is the worst possible you could imagine and 6 is exactly as you would like to be. Respondents are then asked to “spend” 10 points to show which areas of their life they feel are most important to their overall QoL. In our study, participants were asked to spend 10,000 Uganda shillings, which they could relate to more easily than 10 points. They did not have to spend their shillings on all five items but they had to spend all the shillings. The scores are aggregated and transformed to a scale of 0–100. A higher score indicates a better global QoL. A cutoff of 60 was used to determine high global QoL (≥60) and low global QoL (<60) [36].

The Center for Epidemiological Depression Scale (CES-D) [37] was used to screen for depression. The CES-D has been used widely in Uganda [38, 39]. A cutoff total score of ≥16 is used to indicate probable depression [37].

The CAGE questionnaire was used to screen for alcoholism. CAGE has been shown to have cross-cultural validity because it does not focus on the specifics of alcohol use [40]. A cutoff of ≥2 was used to indicate probable alcohol abuse. A score of 2 or 3 represents a high index of suspicion of alcoholism, and a score of 4 may be diagnostic for alcoholism [41].

ART adherence was measured using a 3-day self-report and a cutoff of 95 % was used to determine adherence (≥95 %) and non-adherence (<95 %). Self-reporting has been shown to have adherence levels not different from unannounced pill count, visual analog scale and electronic drug monitoring [42]. A cutoff of 95 % adherence has also been associated with better viral suppression [43, 44].

### Statistical analysis

The main outcome was QoL using the subscales from the MOS-HIV as PHS and MHS, and global QoL measured by the GPGI. The MOS-HIV was scored using the guidelines obtained from the Mapi Research Trust, and missing data were handled as per the measurement guidelines [28, 29, 33]. Descriptive characteristics were analyzed and presented as percentages, means with standard deviations, medians, and interquartile ranges. The CD4 count change was obtained as the difference between the 6-month and baseline CD4 counts as the main predictor variable. Univariate analysis was done for PHS and MHS and CD4 change using linear generalized estimating equations (GEE) among patients on ART and those not on ART separately. In a multivariate linear model, we controlled for baseline CD4, sociodemographic characteristics, behavioral characteristics, and other clinical characteristics that have been shown to be associated with QoL, such as WHO disease stage and depression (confounders were selected from previous studies on the basis of clinical, epidemiological and biological relevance). Depression was not added as a covariate in the MHS models to avoid collinearity because the MHS partly assesses for depression. Logistic GEE were used to assess the association between GPGI score and change in CD4 count in the univariate and multivariate analyses, controlling for the potential confounders listed above. The analysis was done separately for patients on ART and those that did not receive ART. The interaction between CD4 change and study visit as a measure of time was tested for significance. All statistical analyses were conducted using Stata statistical software 12.1 (StataCorp LP, College Station, TX, USA). Statistical significance was defined as a *p*-value <0.05. The interviews were transcribed and translated for coding and thematic analysis. Data were analyzed manually using a framework approach to thematic analysis.

### Ethics

The study was approved by Makerere University College of Health Sciences Higher Degrees Research and Ethics Committee and the Uganda National Council for Science and Technology (UNCST, Ref HS 1161). All participants provided written, informed consent prior to participation in the study. Participants were informed that refusal to participate would not affect their care services in any way. No personal identifiers such as names were collected. Confidentiality was maintained throughout the study, from the data collection process to storage and analysis of the data.

## Results

Of the 1274 PLHIV enrolled in the study, 115 (9 %) were lost to follow-up by the 6-month visit. Of the patients lost to follow-up, 54 (47 %) were on ART; there were no differences in the attrition factors (died, lost, and changed care facility) between patients on ART and those not on ART. The total used for this analysis was 1159 patients who had both baseline and 6-month CD4 counts. The majority of these were women 823 (71 %), and 586 (51 %) were on ART. The majority of the patients were less than 30 years old 482 (41 %). Only 322 (28 %) of the total 1159 participants reported using alcohol; 184 (57 %) were probable alcohol abusers with a CAGE score ≥2. Table 1 has the details of the socio-demographic characteristics.

**Table 1:** Socio-demographic characteristics of 1159 study participants at the Mulago HIV clinic in Uganda between April 2012 and December 2013

Characteristic	Whole sample	ART	No ART
	*n* = 1159 (%)	*n* = 586 (%)	*n* = 573 (%)
Gender			
Female	823 (71)	386 (66)	436 (76)
Male	336 (29)	200 (34)	137 (24)
Education level			
≤ Primary	594 (51)	298 (51)	296 (52)
Secondary	458 (40)	232 (40)	226 (39)
Apprenticeship	83 (7)	42 (7)	41 (7)
Tertiary	24 (2)	14 (2)	10 (2)
Age in years			
<30	482 (41)	255 (44)	227 (40)
30–39	426 (37)	212 (36)	214 (37)
≥40	251 (22)	119 (20)	132 (23)
Monthly income (USD)			
<20	362 (31)	180 (31)	182 (32)
20–60	329 (29)	154 (26)	175 (30)
>60	468 (40)	252 (43)	216 (38)
Religion			
Christian	951 (82)	476 (81)	475 (83)
Muslim	187 (16)	99 (17)	88 (15)
Other	21 (2)	11 (2)	10 (2)
Marital status			
Currently married	703 (61)	352 (60)	351 (61)
Divorced/separated	274 (24)	144 (24)	130 (23)
Single	76 (6)	45 (8)	31 (5)
Widowed	106 (9)	45 (8)	61 (11)
Employed			
Yes	938 (81)	478 (82)	460 (80)
No	221 (19)	108 (18)	113 (20)
Social support			
Yes, family	920 (79)	488 (83)	432 (75)
Yes, other	194 (17)	80 (14)	114 (20)
None	45 (4)	18 (3)	27 (5)
Alcohol consumption			
Yes	322 (28)	141 (24)	181 (32)
No	837 (72)	445 (76)	392 (68)
Smoking			
Yes	50 (4)	23 (4)	27 (5)
No	1109 (96)	563 (96)	546 (95)

Abbreviation: ART, antiretroviral therapy

The median baseline CD4 count was 396 cells/μL (IQR 260–634) and 501 at 6 months (IQR 381–635). Among individuals on ART, 545 (93 %) had an adherence score of ≥95 % at the 3- and 6-month visits. Overall, 313 (27 %) participants had an opportunistic infection at baseline and 220 (19 %) at 6 months. Details of other clinical characteristics are given in Table 2.

**Table 2:** Clinical characteristics of 1159 study participants at the Mulago HIV clinic in Uganda between April 2012 and December 2013

Characteristic	Whole sample	ART	No ART
	*n* = 1159 (%)	*n* = 586 (%)	*n* = 573 (%)
WHO disease stage			
Stages I and II	962 (83)	470 (80)	492 (86)
Stages III and IV	197 (17)	116 (20)	81 (14)
Opportunistic infection			
Yes	310 (27)	187 (32)	123 (21)
No	849 (73)	399 (68)	450 (79)
Depression screening, baseline score			
No depression (<16)	777 (67)	380 (65)	397 (69)
Probable depression (≥16)	382 (33)	206 (35)	176 (31)
Depression screening score, 6 months			
No depression (<16)	845 (73)	443 (76)	402 (70)
Probable depression (≥16)	314 (27)	143 (24)	171 (30)
Baseline CD4 count (cells/μL)			
<100	104 (9)	104 (18)	0 (0)
101–350	436 (38)	436 (74)	0 (0)
>350	619 (53)	46 (8)	573 (100)
CD4 count at 6 months			
<100	27 (2)	26 (4)	1 (0)
101–350	205 (18)	179 (31)	26 (5)
>350	927 (80)	381 (65)	546 (95)

Abbreviation: ART, antiretroviral therapy

The MOS-HIV subscales used to construct the PHS and MHS at baseline, month 3 and month 6 ranged from pain with a mean of 26.4 (SD 23.4) to social functioning with a mean of 93.0 (SD 18.8), pain 21.2 (18.6) to social functioning 96.0 (13.5), pain 20.4 (17.9) to social functioning 96.1 (31.6) at baseline, month 3 and month 6 respectively. Table 3 has details of all the subscales. These were transformed means to a scale of 0–100 to make easy comparisons with other MOS-HIV Survey data.

**Table 3:** Summary of the MOS HIV subscales of 1274 study participants used to construct the PHS and MHS at baseline, month three and month six at the Mulago HIV clinic, 2013

Subscale	Range of raw scores	Transformed mean score (SD) at baseline	Transformed mean score (SD) at Month 3	Transformed mean score (SD) at Month 6
General Health	5–25	53.4 (13.3)	52.7 (11.8)	53.2 (11.5)
Physical Health	6–18	86.3 (16.8)	89.7 (15.9)	90.3 (15.3)
Role Function	2–4	67.1 (37.5)	72.9 (32.7)	74.0 (31.6)
Social Function	1–6	93.0 (18.8)	96.0 (13.5)	96.1 (13.1)
Cognitive Function	4–24	81.3 (16.9)	86.3 (14.6)	87.2 (14.6)
Pain	2–11	26.5 (23.7)	21.2 (18.6)	20.4 (17.9)
Mental Health	5–30	55.5 (7.9)	54.7 (6.4)	54.3 (6.6)
Vitality	4–24	49.5 (8.2)	48.2 (6.4)	48.2 (6.4)
Health Distress	4–24	86.0 (22.1)	93.1 (15.7)	93.6 (15.6)
Quality of life	1–5	47.4 (26.3)	42.6 (24.1)	40.2 (24.5)
Health Transition	1–5	47.4 (26.3)	42.6 (24.1)	40.2 (24.5)

Abbreviation: SD, standard deviation

Baseline visit there were 1274 patients, month three 1217 and month six 1159 patients and data was collected from April 2012 to December 2013

The baseline mean PHS was 46.6 (SD 4.2), MHS 46.6 (SD 4.2) and median GPGI 71.7 (IQR 56.7–85.0) Table 3. No statistically significant change in CD4 count over time was found when testing for interaction among the three QoL outcomes (PHS *p* = 0.440, MHS *p* = 0.245 and GPGI score OR = 1 *p* = 0.030) indicating similar patterns of change over time in both groups. Table 4 summarizes the PHS, MHS and GPGI scores at all three visits as means (SD) and medians (IQR).

**Table 4:** Summary of QoL scores of study participants at the three study visits attending the Mulago HIV clinic in Uganda between April 2012 and December 2013 indicated as mean (SD) or median (IQR)

Summary measures	Whole sample	ART	NO ART
PHS	Mean (SD)	Mean (SD)	Mean (SD)
Baseline visit	46.6 (4.2)	46.5 (4.5)	46.6 (3.9)
Month 3 visit	46.9 (4.0)	47.0 (3.9)	46.9 (4.0)
Month 6 visit	47.1 (3.8)	47.2 (3.8)	47.0 (3.9)
MHS	Mean (SD)	Mean (SD)	Mean (SD)
Baseline visit	46.9 (4.2)	46.4 (4.5)	47.4 (3.8)
Month 3 visit	47.5 (3.4)	47.3 (3.5)	47.7 (3.4)
Month 6 visit	47.4 (3.4)	47.1 (3.4)	47.6 (3.3)
GPGI	Median (IQR)	Median (IQR)	Median (IQR)
Baseline visit	71.7 (56.7–85.0)	71.6 (55.0–85.0)	73.3 (56.7–86.7)
Month 3 visit	73.3 (56.7–85.0)	71.7 (56.7–85.0)	75.0 (56.7–86.7)
Month 6 visit	73.3 (60.0–85.0)	73.3 (58.3–85.0)	73.3 (60.0–85.0)

Abbreviations: QoL, quality of life; SD, standard deviation; IQR, interquartile range; PHS, Physical Health Score; MHS, Mental Health Score; GPGI, Global Person Generated Index

Sample size 1274 at baseline (ART *n* = 640, no ART *n* = 634), 1217(ART *n* = 625, no ART = 592) at month 3 and 1159 (ART *n* = 586, no ART *n* = 576) at month 6, data collected from April 2012 to December 2013 at the Mulago HIV clinic in Uganda

There was no significant association between change in CD4 count and PHS, MHS and GPGI QoL scores in the univariate analysis and after adjusting for potential confounders of PHS and GPGI scores among patients on ART. Confounders included baseline CD4 count, study visit, gender, age, education level, marital status, alcohol consumption, monthly income, smoking status, diagnosis of depression, social support, presence of opportunistic infection, WHO disease stage, religion, and employment status. For MHS, the potential confounders were as above, but without the depression screening score. Change in CD4 count and PHS, MHS and GPGI after adjusting for potential confounders were not significant. Low education level, probable depression and WHO stage III and IV were associated with low PHS, females had lower MHS and MHS improved with time and alcohol consumption and probable depression were also related to low GPGI. Detailed results are presented in Table 4. There was no evidence of multicollinearity; the standard errors (SE) for change in CD4 were the same in the univariate and multivariate analyses for PHS, MHS and GPGI score (Table 5).

**Table 5:** Comparison of change in CD4 count and QoL among 586 patients receiving ART at the Mulago HIV clinic in Uganda between April 2012 and December 2013

Outcome QoL score	PHS		MHS		GPGI	
Predictor variable β coefficient (95 % CI)/ odds ratio (95 % CI)	Univariate	Multivariate	Univariate	Multivariate	Univariate	Multivariate
Change in CD4 count	0.0001 (−0.0013 to 0.0016)	0.0001 (−0.0014 to 0.0015)	0.0004 (−0.0009 to 0.0018)	0.0009 (−0.0005 to 0.0023)	0.9999 (0.9990 to 1.0001)	0.9999 (0.9991 to 1.0008)
	SE = 0.0007	SE = 0.0007	SE = 0.0007	SE = 0.0007	SE = 0.0004	SE = 0.0004
Baseline CD4 count (cells/μL)						
<100	Ref	Ref	Ref	Ref	Ref	Ref
101−350	0.82 (0.21 to 1.44)	0.64 (0.02 to 1.25)	0.12 (−0.44 to 0.68)	−0.02 (−0.61 to 0.57)	0.89 (0.63 to 1.24)	0.79 (0.54 to 1.14)
>350	0.90 (0.03 to 1.76)	0.62 (−0.23 to 1.47)	0.32 (−0.47 to 1.11)	0.26 (−0.56 to 1.08)	0.81 (0.51 to 1.30)	0.68 (0.41 to 1.13)
Gender						
Male	Ref	Ref	Ref	Ref	Ref	Ref
Female	−0.30 (−0.78 to 0.18)	0.02 (−0.50 to 0.54)	−0.87 (−1.30 to −0.43)	−0.78 (−1.28 to −0.29)	0.83 (0.64 to 1.07)	1.14 (0.84 to 1.56)
Age in years						
<30	Ref	Ref	Ref	Ref	Ref	Ref
30−39	−0.46 (−0.98 to 0.053)	−0.44 (−0.95 to 0.07)	0.46 (−0.01 to 0.93)	0.27 (−0.23 to 0.76)	0.92 (0.70 to 1.20)	0.91 (0.67 to 1.22)
≥40	−0.56 (−1.17 to 0.05)	0.50 (−1.12 to 0.11)	0.15 (−0.40 to 0.71)	0.10 (−0.49 to 0.69)	0.93 (0.67 to 1.30)	0.95 (0.66 to 1.38)
Education level						
≤Primary	Ref	Ref	Ref	Ref	Ref	Ref
Secondary	0.69 (0.21 to 1.17)	0.44 (−0.03 to 0.92)	−0.19 (−0.63 to 0.25)	−0.11 (−0.56 to 0.35)	1.27 (0.98 to 1.65)	1.12 (0.84 to 1.48)
Apprenticeship	1.74 (0.81 to 2.66)	0.96 (0.06 to 1.86)	0.38 (−0.47 to 1.23)	0.23 (−0.63 to 1.09)	1.17 (0.74 to 1.84)	0.84 (0.53 to 1.39)
Tertiary	2.31 (0.94 to 3.68)	1.72 (0.28 to 3.15)	−1.05 (−2.31 to 0.21)	−1.86 (−3.23 to −0.48)	3.72 (1.17 to 11.87)	3.65 (1.06 to 12.56)
Marital status						
Currently married	Ref	Ref	Ref	Ref	Ref	Ref
Divorced/separated	−0.15 (−0.70 to 0.40)	0.15 (−0.40 to 0.70)	−0.60 (−1.10 to −0.10)	−0.42 (−0.95 to 0.10)	0.73 (0.54 to 0.97)	0.73 (0.54 to 0.99)
Single	0.53 (−0.34 to 1.39)	0.58 (−0.31 to 1.46)	−0.68 (−1.46 to 0.10)	−0.52 (−1.37 to 0.33)	0.92 (0.58 to 1.44)	0.85 (0.51 to 1.42)
Widowed	−0.59 (−1.45 to 0.27)	−0.41 (−1.27 to 0.46)	−0.73 (−1.51 to 0.05)	−0.49 (−1.31 to 0.33)	0.63 (0.40 to 0.99)	0.73 (0.45 to 1.18)
Alcohol consumption						
Yes	Ref	Ref	Ref	Ref	Ref	Ref
No	−0.15 (−0.68 to 0.39)	0.02 (−0.51 to 0.55)	−0.20 (−0.69 to 0.28)	−0.27 (−0.77 to 0.24)	1.49 (1.13 to 1.96)	1.56 (1.14 to 2.14)
Income per month in USD						
<20	Ref	Ref	Ref	Ref	Ref	Ref
20−60	−0.22 (−0.82 to 0.37)	−0.18 (−0.91 to 0.55)	−0.12 (−0.67 to 0.43)	−0.47 (−1.17 to 0.23)	1.00 (0.73 to 1.37)	0.99 (0.65 to 1.50)
>60	0.77 (0.24 to 1.31)	0.37 (−0.33 to 1.06)	0.27 (−0.23 to 0.76)	−0.26 (−0.93 to 0.41)	1.59 (1.20 to 2.12)	1.43 (0.95 to 2.14)
Smoking status						
Yes	Ref	Ref	Ref	Ref	Ref	Ref
No	−0.40 (−1.61 to 0.80)	−0.27 (−1.46 to 0.92)	0.43 (−0.66 to 1.52)	0.78 (−0.35 to 1.92)	1.33 (0.73 to 2.42)	1.04 (0.50 to 2.18)
Social support						
Yes, family	Ref	Ref	Ref	Ref	Ref	Ref
Yes, other	0.52 (−0.14 to 1.19)	0.49 (−0.15 to 1.14)	−0.19 (−0.80 to 0.41)	−0.10 (−0.72 to 0.51)	0.83 (0.60 to 1.15)	0.98 (0.69 to 1.39)
None	−0.37 (−1.72 to 0.97)	−0.44 (−1.76 to 0.87)	−1.27 (−2.49 to −0.04)	−1.15 (−2.41 to 0.10)	0.74 (0.41 to 1.34)	0.84 (0.44 to 1.63)
Depression score No depression						
(<16)	Ref	Ref	-	-	Ref	Ref
Probable depression (≥16)	−1.95 (−2.35 to −1.55	−1.71 (−2.12 to −1.31)			0.37 (0.30 to 0.45)	0.39 (0.31 to 0.49)
Opportunistic infection						
Yes	Ref	Ref	Ref	Ref	Ref	Ref
No	0.46 (−0.03 to 0.96)	0.07 (−0.42 to 0.56)	0.30 (−0.15 to 0.75)	0.13 (−0.34 to 0.59)	1.33 (1.03 to 1.72)	1.24 (0.94 to 1.64)
WHO stage						
Stages I and II	Ref	Ref	Ref	Ref	Ref	Ref
Stages III and IV	−1.09 (−1.66 to −0.52)	−0.76 (−1.33 to −1.19)	−0.34 (−0.86 to 0.19)	−0.24 (−0.78 to 0.31)	0.76 (0.57 to 1.02)	0.78 (0.57 to 1.07)
Religion						
Christian	Ref	Ref	Ref	Ref	Ref	Ref
Muslim	−0.41 (−1.02 to 0.20)	−0.17 (−0.77 to 0.43)	0.48 (−0.08 to 1.03)	0.55 (−0.02 to 1.13)	1.10 (0.79 to 1.53)	1.11 (0.78 to 1.59)
Other	0.03 (−1.02 to 0.20)	−0.20 (−1.79 to 1.38)	−0.70 (−2.21 to 0.81)	−0.71 (−2.22 to 0.81)	0.89 (0.41 to 1.90)	0.76 (0.36 to 1.59)
Employment status						
Yes	Ref	Ref	Ref	Ref	Ref	Ref
No	−0.31 (−0.90 to 0.28)	−0.12 (−0.90 to 0.65)	−0.27 (−0.81 to 0.26)	−0.39 (−1.13 to 0.35)	0.78 (0.58 to 1.05)	0.97 (0.63 to 1.51)

Abbreviations: 95 % CI, 95 % confidence interval; SE, standard error

Among patients not receiving ART, there was no significant association between change in CD4 count and PHS and MHS in the univariate and multivariate analyses. There was a negative association between GPGI score and change in CD4 count with OR = 0.9991 in the univariate analysis, and OR = 0.9990 in the multivariate analysis for high GPGI with a unit change in CD4. The associations were very small (0.09 %,) in univariate analysis and 0.1 %) in multivariate analysis. Older age, probable depression were associated with low PHS, low income and presence of opportunistic infection were associated with low MHS and older age, low education and probable depression were also negatively associated with GPGI (Table 6).

**Table 6:** Comparison of change in CD4 count and QoL among 573 patients not receiving ART at the Mulago HIV clinic in Uganda between April 2012 and December 2013

Outcome QoL score	PHS		MHS		GPGI	
Predictor variable β coefficient/Odds Ratio (95 % CI)	Univariate	Multivariate	Univariate	Multivariate	Univariate	Multivariate
Change in CD4 count	−0.0003 (−0.0015 to 0.0010)	−0.0003 (−0.0011 to 0.0008)	−0.0001 (−0.0012 to 0.0009)	0.0000 (−0.0010 to 0.0011)	0.9991 (0.9983 to 0.9998)	0.9990 (0.9982 to 0.9997)
	SE = 0.0006	SE = 0.0006	SE = 0.0005	SE = 0.0005	SE = 0.0004	SE = 0.0004
Gender						
Male	Ref	Ref	Ref	Ref	Ref	Ref
Female	−0.56 (−1.11 to −0.14)	−0.71 (−1.33 to −0.09)	−0.13 (−0.58 to 0.33)	0.07 (−0.46 to 0.61)	1.13 (0.84 to 1.52)	1.26 (0.89 to 1.77)
Age in years						
<30	Ref	Ref	Ref	Ref	Ref	Ref
30−39	−0.21 (−0.73 to 0.32)	−0.32 (−0.86 to 0.22)	0.39 (−0.05 to 0.83)	0.38 (−0.08 to 0.84)	0.78 (0.58 to 1.05)	0.79 (0.58 to 1.09)
≥40	−1.16 (−1.77 to −0.55)	−1.35 (−2.00 to −0.70)	0.29 (−0.22 to 0.81)	0.17 (−0.39 to 0.73)	0.62 (0.45 to 0.87)	0.62 (0.43 to 0.89)
Education level						
≤Primary	Ref	Ref	Ref	Ref	Ref	Ref
Secondary	0.74 (0.24 to 1.23)	0.66 (0.16 to 1.15)	0.12 (−0.29 to 0.54)	0.11 (−0.02 to 0.53)	1.51 (1.15 to 1.97)	1.26 (0.96 to 1.66)
Apprenticeship	0.96 (0.04 to 1.87)	0.46 (−0.48 to 1.40)	−0.03 (−0.80 to 0.73)	−0.03 (−0.83 to 0.78)	1.75 (1.03 to 2.98)	1.50 (0.86 to 2.63)
Tertiary	1.33 (−0.59 to 3.25	0.82 (−1.03 to 2.67)	−0.11 (−1.73 to 1.50)	0.06 (−1.54 to 1.65)	11.85 (1.73 to 80.95)	10.11 (1.52 to 67.14)
Marital status						
Currently married	Ref	Ref	Ref	Ref	Ref	Ref
Divorced/separated	0.55 (−0.52 to 0.63)	0.22 (−0.36 to 0.81)	0.21 (−0.27 to 0.69)	0.22 (−0.28 to 0.72)	0.82 (0.60 to 1.12)	0.84 (0.61 to 1.15)
Single	−0.03 (−1.07 to 1.02)	−0.51 (−1.58 to 0.57)	−0.31 (−1.18 to 0.55)	−0.35 (−1.27 to 0.57)	1.07 (0.56 to 2.05)	0.82 (0.43 to 1.56)
Widowed	−0.26 (−1.05 to 0.53)	0.45 (−0.36 to 1.27)	−0.02 (−0.68 to 0.63)	0.11 (−0.60 to 0.81)	0.78 (0.52 to 1.17)	0.86 (0.54 to 1.38)
Alcohol consumption						
Yes	Ref	Ref	Ref	Ref	Ref	Ref
No	−0.23 (−0.73 to 0.28)	−0.02 (−0.52 to 0.49)	0.01 (−0.41 to 0.43)	0.10 (0.34 to 0.53)	0.93 (0.71 to 1.22)	0.94 (0.70 to 1.26)
Income per month in USD						
<20	Ref	Ref	Ref	Ref	Ref	Ref
20−60	0.36 (−0.24 to 095)	0.41 (−0.30 to 1.11)	−0.22 (−0.72 to 0.27)	−0.66 (−1.27 to −0.06)	1.28 (0.93 to 1.77)	1.18 (0.82 to 1.70)
>60	0.79 (0.23 to 1.35)	0.54 (−0.16 to 1.25)	−0.06 (−0.53 to 0.40)	−0.43 (−1.03 to 0.18)	1.60 (1.18 to 2.17)	1.33 (0.91 to 1.95)
Smoking status						
Yes	Ref	Ref	Ref	Ref	Ref	Ref
No	0.64 (−0.48 to 1.75)	0.27 (−0.88 to 1.42)	−0.72 (−1.64 to 0.20)	−0.87 (−1.85 to 0.12)	2.14 (1.19 to 3.84)	1.62 (0.91 to 2.89)
Depression score No depression						
(<16)	Ref	Ref	-	-	Ref	Ref
Probable depression (≥16)	−1.07 (−1.46 to −0.67)	−1.01 (−1.41 to −0.60)			0.41 (0.33 to 0.52)	0.43 (0.33 to 0.55)
Social support						
Yes, family	Ref	Ref	Ref	Ref	Ref	Ref
Yes, other	0.063 (−0.53 to 0.66)	0.003 (−0.60 to 0.60)	0.28 (−0.21 to 0.77)	0.41 (−0.11 to 0.92)	0.91 (0.67 to 1.24)	0.96 (0.69 to 1.33)
None	−0.01 (−1.12 to 1.10)	−0.01 (−1.10 to 1.07)	0.70 (−0.21 to 1.61)	0.65 (−0.28 to 1.58)	1.10 (0.62 to 1.95)	1.20 (0.65 to 2.20)
Opportunistic infection						
Yes	Ref	Ref	Ref	Ref	Ref	Ref
No	0.41 (−0.15 to 0.97)	0.22 (−0.36 to 0.79)	0.59 (0.12 to 1.06)	0.66 (0.17 to 1.16)	1.06 (0.78 to 1.45)	1.02 (0.73 to 1.42)
WHO stage						
Stages I and II	Ref	Ref	Ref	Ref	Ref	Ref
Stages III and IV	−0.63 (−1.30 to 0.04)	−0.51 (−1.19 to 0.17)	0.38 (−0.94 to 0.18)	−0.19 (−0.77 to 0.40)	0.86 (0.59 to 1.25)	1.00 (0.68 to 1.45)
Religion						
Christian	Ref	Ref	Ref	Ref	Ref	Ref
Muslim	−0.74 (−1.39 to −0.09)	−0.30 (−0.96 to 0.37)	0.27 (−0.27 to 0.81)	0.24 (−0.33 to 0.82)	1.03 (0.71 to 1.50)	1.19 (0.80 to 1.78)
Other	−0.55 (−2.42 to 1.31)	−0.57 (−2.35 to 1.21)	1.13 (−0.42 to 2.68)	1.40 (−0.13 to 2.94)	1.11 (0.45 to 2.78)	0.82 (0.28 to 2.46)
Employment status						
Yes	Ref	Ref	Ref	Ref	Ref	Ref
No	−0.28 (−0.87 to 0.31)	0.26 (−0.48 to 1.00)	−0.23 (−0.72 to 0.26)	−0.39 (−1.02 to 0.25)	0.70 (0.51 to 0.97)	0.73 (0.49 to 1.09)

Abbreviations: 95 % CI, 95 % confidence interval; SE, standard error

The in-depth interviews revealed other factors to be important for a good QoL in addition to good health, such as having money, building a house, family relations with spouses and children. The themes identified were “liveabilty of environment (work and finances), Life ability (health, treatment, stigma, depression), utility of life (goals/expectations, family relations) and appreciation of life (happiness) [26]. However, they still suffered from stigma, depression and lack of disclosure of their HIV status details of the qualitative results have been published elsewhere [26].

## Discussion

Our evaluation provides evidence that there is no significant association between changes in CD4 count and changes in PHS, MHS and GPGI QoL scores among patients on ART and those not on ART. The change in GPGI score was too small for change in CD4 count to be considered a predictor of change in QoL [45]. Contrary to our expectations, we found no positive association between change in CD4 count and QoL among patients on ART and no expected association between decreased CD4 count and decreased QoL among patients not on ART.

The lack of association between CD4 change and QoL scores is possibly because the majority of the participants were asymptomatic with relatively high CD4 counts, with only 104 (9 %) of patients with counts less than 100 cells/ μL at baseline and 27 (2 %) at 6 months. Therefore, because most participants had a high CD4 count, they had a good QoL. There may be insufficient change in their CD4 count to make a significant change in their QoL. In earlier studies [6, 9], ART was initiated in patients who were quite ill; thus, the change in CD4 count could easily be related to improvement in QoL because patients went from experiencing severe ill-health to rapid recovery of health and functioning, which are likely to impact QoL. Because most of our patients had a high CD4 count (>200), they were relatively well and any increase in CD4 count may not have translated to better QoL. Also, the global QoL in this study population was high in all categories, with IQR between 60 and 85. A score range of 60–80 on subjective measures has been reported as normal QoL [36], so there was not much room for change. The overall mean PHS and MHS in our study are comparable to what has been recently reported among Ugandan and Kenyan patients, with mean PHS of 44.9 and mean MHS of 46.2 [12]. Interestingly, that study reported a positive association between CD4 count and QoL; however it was a cross-sectional analysis with a sample size of 1337 participants and no temporal association compared with our longitudinal study. In addition, their analysis combined patients on ART and those that were not on ART.

Another possible reason for our results is that QoL is a subjective construct and there are many other factors that come into play other than immunological markers. Patients have goals and expectations for QoL that change with time, and clinicians cannot assume that an increase in CD4 count will automatically lead to a better QoL for patients. Our qualitative data showed that QoL as well as general well-being were influenced by income, relationships, emotional well-being and health status [26]. There may also be a role played by other factors, such as socioeconomic status and behavioral characteristics. In the multivariate analysis depression was negatively associated with QoL and this has been reported in other settings [46], Older age has also been associated with poor QoL similar to what was reported in our study [47, 48]. Females patients similar to our study findings have also been reported to have poor QoL compared to males among Ethiopians [49] and other factors such as income and level of education have also been reported to be associated with QoL among PLHIV in both developed and developing countries [5, 7, 13, 50] comparable to the findings from our study. QoL is multifaceted and highly subjective. QoL, response to HIV diagnosis, and treatment trajectory are not linear processes [51] and many other factors contribute to the outcome. Studies of other chronic diseases such as asthma have reported that patients’ “valued life activities” are more strongly associated with changes in QoL than traditional clinical and functional measures [52]. Harding and others also reported that spiritual and social problems affected patients living with HIV in East Africa rather than physical challenges [12].

Our finding of weak to no correlation between change in CD4 count and QoL scores is similar to findings of other studies carried out in sub-Saharan Africa [17, 18]. The basic characteristics of our study sample were similar to the populations in those studies. The majority of participants were females, aged mid-30s with low educational level, which may be the reason for our similar results. However, one of the above studies included both not on ART and ART-experienced patients [17] and also was a secondary data analysis of 642 patients. In the other study, although participants had CD4 counts lower than 250 cells/μL, they were not on ART; in addition, it was a cross-sectional study with only 90 participants [18]. Likewise, another cross-sectional study in Tanzania reported no association between change in CD4 count and QoL among 329 patients who had been on ART for more than 6 months [20]. The change in CD4 count in that population was the difference between baseline CD4 count and the most recent CD4 count. The time between these counts varied and QoL was measured at one time point. However, Weinfurt and colleagues found a positive association between change in CD4 count and QoL and reported CD4 count to be a strong long-term predictor of QoL [16]. That was a double randomized clinical trial and participants were on ART and followed for a period of 2 years. In the above study, participants were aware of their CD4 levels at the time of interview, which could have influenced their MOS-HIV QoL ratings. They also received ART regimens different to those of our study group; they received either didanosine or didanosine plus delavirdine mesylate. This was before highly active antiretroviral therapy (HAART) became available. Besides, it may not be plausible to compare results from a randomized clinical trial to our results from a cohort study. Other studies conducted within the HAART era have also shown some improvements in viral loads and CD4 counts [53, 54]. These studies did not directly measure the association between QoL and CD4 counts, and participants had advanced disease. Call and colleagues also reported CD4 count to be a strong predictor of the following subscales on the SF-36 quality of life measure: physical component summary, physical functioning, role function and general health [24]. The 158 participants in that study were followed for a period of 12 months. They were enrolled if they were initiating ART or changing ART regimen. Patients who change ART, most likely after failing on the first therapy, are more likely to positively respond with the new therapy both immunologically and in terms of improved QoL.

This study has limitations. The 6-month follow-up for chronic illness may be rather short; a longer follow-up time is recommended. This was not a randomized study, and viral load testing could have enriched this study as a more accurate measure of disease burden. The large sample size may have caused a weak association with GPGI.

Nevertheless, the strengths of this study include the large sample size and longitudinal design to specifically assess the association between immunological markers and QoL. To our knowledge this is the first study to look at this in sub-Saharan Africa. Most importantly, confounders of QoL were statistically controlled for. The results of the qualitative sub-study, which provided insight into the context of participants’ lives, help to explain why CD4 count alone cannot be used to predict QoL.

## Conclusions

These data suggest that an increase or decrease in CD4 may not translate to better or worse QoL within a period of 6 months among PLHIV with relatively high CD4 count. It may therefore be paramount for clinicians and policy makers to consider other factors, such as income and psychosocial support, in monitoring QoL among HIV/AIDS patients and not to rely on change in immunological markers alone. HIV/AIDS is now an incurable chronic condition; therefore, a good QoL together with prolonged survival is paramount.

## Recommendation

In addition to monitoring immunological markers, other psychosocial and socioeconomic factors should be underscored in management of HIV patients in order to optimize treatment outcomes.

A similar study with longer follow-up period would further enrich the findings of this study.

## Abbreviations

QoL: quality of life
ART: antiretroviral therapy
OR: odds ratio
CI: confidence interval
PLHIV: people living with HIV
MHS: Mental Health Score
PHS: Physical Health Score
GPGI: Global Person Generated Index
MOS-HIV: Medical Outcomes Survey HIV
SD: standard deviation
IQR: interquartile range
HAART: highly active antiretroviral therapy
